# The viral load monitoring cascade in HIV treatment programmes in sub-Saharan Africa: a systematic review

**DOI:** 10.1186/s12889-024-20013-x

**Published:** 2024-09-27

**Authors:** Annalise Kippen, Londiwe Nzimande, Dickman Gareta, Collins Iwuji

**Affiliations:** 1 Brighton and Sussex Medical School, Brighton, UK; 2 Africa Health Research Institute, KwaZulu-Natal, South Africa; 3 Department of Global Health and Infection, Brighton and Sussex Medical School, University of Sussex, Brighton, UK

**Keywords:** HIV, Viral load monitoring, Sub-Saharan Africa, Failure cascade, Decentralisation

## Abstract

**Background:**

The United Nations’ 95-95-95 (95% of people with HIV being aware of their diagnosis, 95% of those aware of their diagnosis being on treatment and 95% achieving viral suppression) target aims to reduce morbidity and mortality of HIV. However, with 60% of new HIV infections occurring in sub-Saharan Africa (SSA), achieving this target in the region is challenging. Viral load (VL) monitoring is the gold-standard approach of assessing treatment efficacy, and its implementation into national health systems is a global health priority if elimination of HIV as a public health threat is to be achieved by 2030. This systematic review aims to investigate VL monitoring outcomes in SSA, and to identify gaps and possible interventions to help nations meet their 2030 targets.

**Methods:**

A literature search of three electronic platforms (MEDLINE, EMBASE and Global Health) was undertaken from 1 January to 9 August 2024 to identify studies published in English and conducted in SSA. The primary outcome was the proportion of people living with HIV (PLHIV) on antiretroviral therapy (ART) with routine VL monitoring at the recommended time points (initially, 6 months, 12 months and annually). Secondary outcomes reported proportions of PLHIV who received routine VL monitoring who went on to complete the cascade of care after identified virological failure [enhanced adherence counselling (EAC), switch to second-line ART, and finally viral suppression].

**Results:**

The initial search identified 342 papers, of which 35 studies were included for narrative synthesis. Included studies reported on findings from 14 African countries and demonstrated extensive variation in rates of VL monitoring (range: 24.3-99.7%, mean: 63.8%). Results were more unfavourable in the latter steps of the viral load monitoring cascade, with a range of 0-88%, and a switch to second-line ART mean of 42% (range: 4.4-93%). Studies with additional support, and those with community-based models of care, had higher rates of VL testing and viral suppression.

**Conclusions:**

VL monitoring and management of virological failure are suboptimal in many SSA countries due to individual and health system-related challenges. Health system strengthening is vital to ensure the sustainability of HIV treatment programmes and the achievement of 95-95-95 targets by 2030.

**Supplementary Information:**

The online version contains supplementary material available at 10.1186/s12889-024-20013-x.

## Background

The Human Immunodeficiency Virus (HIV) remains a significant cause of morbidity and mortality in many countries, despite significant advances in knowledge of the virus, as well as its treatment and prevention with the development of Antiretroviral Therapy (ART) [1]. The HIV epidemic continues to disproportionately affect the World Health Organisation (WHO) African region, where an estimated 25.9 million people are currently living with the disease, accounting for 65% of global HIV prevalence [2]. To eliminate HIV as a public health threat by 2030 as per the Joint United Nations Programme on HIV and AIDS (UNAIDS) aim, the 90-90-90 campaign was launched in 2014 [3]. This campaign proposed that by 2020, 90% of all people living with HIV (PLHIV) would know their status: 90% of PLHIV with known diagnosis would be treated with ART; 90% of those treated with ART would be virally suppressed, and therefore untransmissible [4]. This equated to 73% of all PLHIV being virally suppressed by 2020, reducing HIV related deaths by 90% worldwide [5]. However, as of 2020, this level of suppression had not yet been achieved by some countries with a new goal of 95-95-95 targets proposed to be achieved by 2030 [6]. A review conducted by Pham et al. in 2022 looked at low- and middle-income countries and their progress to eliminating new HIV infections by 2030 [6]. However, given that as of 2020, 60% of all new HIV infections occurred in sub-Saharan Africa (SSA) [6], a more detailed view on this region would be beneficial to focus public health interventions [6].

**Fig. 1:**
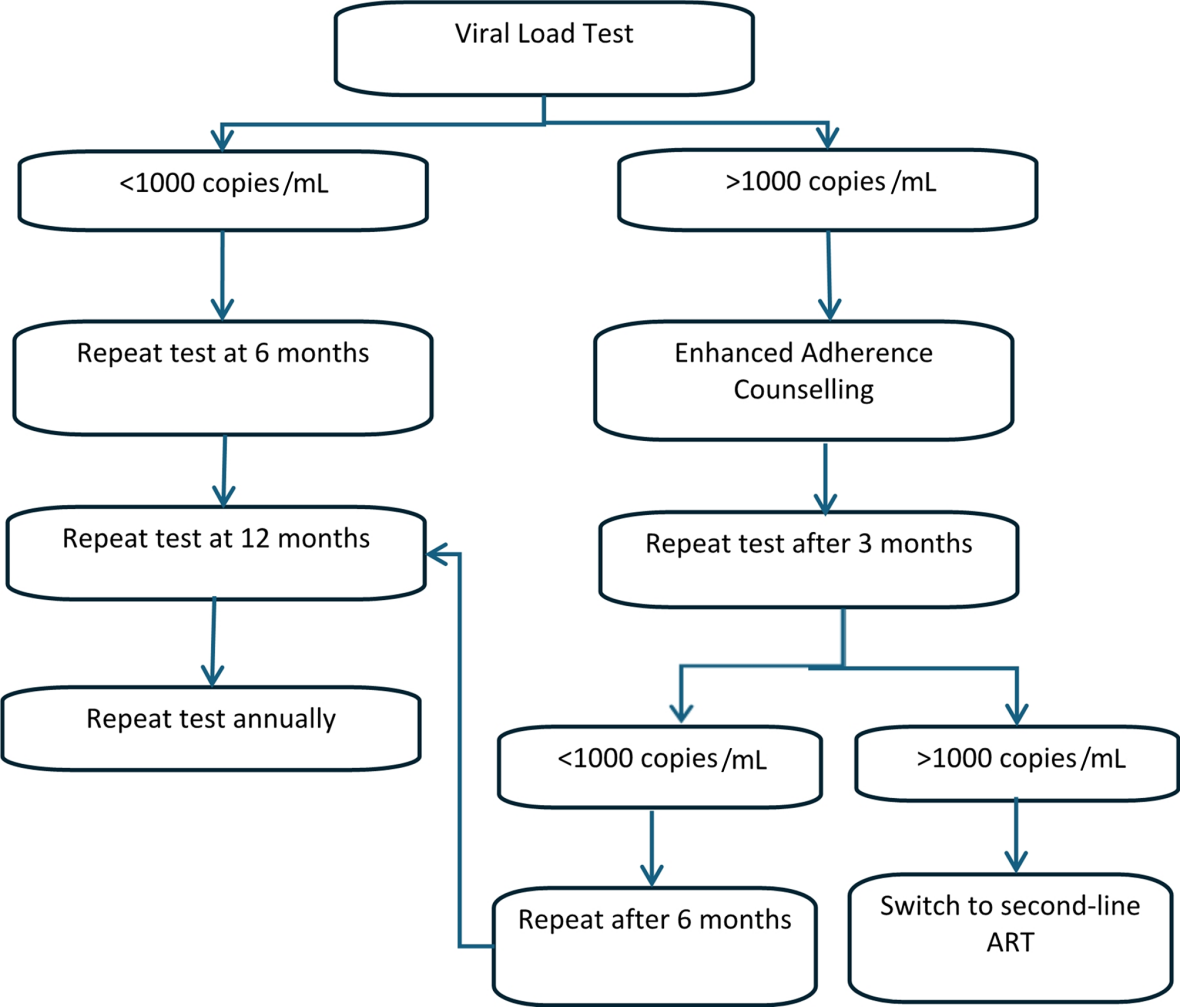
The HIV viral load monitoring cascade and treatment failure cascade

Viral suppression is the most important target in terms of reducing HIV transmission, as well as reducing morbidity and mortality, therefore monitoring all PLHIV for viral suppression is a global health priority [7]. HIV RNA viral load (VL) monitoring is recommended by the WHO as the gold standard approach to assess the success of ART and viral suppression [8]. In 2016, WHO guidelines were revised to recommend VL monitoring over CD4 count, another method of monitoring ART and disease progression [7], as it identifies treatment failure earlier, allowing for less delay in clinical decision-making regarding treatment change [9]. Virological failure is defined by the WHO as having a viral load > 1000 copies/ml [10]. If treatment failure is detected, this should trigger what is known as the “treatment failure cascade”, a set of steps recommended by the WHO, which most LMICs have integrated into national guidelines [11]. The guidelines recommend a VL test after six months on ART, then after 12 months, and annually thereafter. If failure occurs, enhanced adherence counselling (EAC) should be given to the patient, followed by a repeat VL test three months later. If the patient is still experiencing virological failure, the patient should be switched to second line ART, followed by subsequent VL monitoring as per the guidelines [12, 13] (Fig. 1). The ultimate goal of this cascade is viral suppression [13].

Identifying treatment failure earlier through VL monitoring will help to reduce the development and spread of drug resistant HIV [14]. The increase in the incidence of drug resistance has a detrimental effect on the achievement of the 95-95-95 target [15] to improve the HIV continuum of care in SSA [5]. Few countries appear to have the capacity to provide reliable access to VL monitoring with the main barrier being because it is technically demanding, resource intensive and an expensive procedure [11]. Therefore, it is important to review each step of the VL monitoring cascade to ensure the efficacy and effectiveness of the programme and identify gaps that may need to be targeted with interventions [16].

This systematic review aims to synthesise the outcomes of each step of the VL monitoring cascade in SSA and identify research gaps that may require future investigation. It is envisaged that this will informing policy makers and program donors of its value and worth in the fight against HIV and enable countries to undergo targeted health system strengthening to achieve 95-95-95 results.

## Methods

### Search strategy and study selection

This systematic review was reported in accordance with the Preferred Reporting Item for Systematic Reviews and Meta-Analyses (PRISMA) statement (Table S1) [17]. Published literature was obtained from three electronic databases using the OVID platform: MEDLINE, EMBASE and Global Health. The following search terms were used: (HIV OR HIV positive) AND (viral load test* OR viral load monitor*) AND (Sub-Saharan Africa OR “Africa south of the Sahara”) AND (clinical outcome OR treatment outcome OR virologic* suppression OR 90-90-90 OR second-line ART). The identified articles were then imported into Rayyan systematic review screening software on the 9th of August 2024.

Once in Rayyan, duplicate papers were identified and removed. A three-step screening process was then carried out, consisting of an initial title screen, an abstract eligibility assessment, and a final full-text review.

Screening of studies for final selection and review was based on inclusion criteria: (i) involving People Living with HIV/AIDS over 15 years of age receiving or being started on ART; (ii) published in English; (iii) published after 2004 when ART became available in this region; (iv) undertaken in SSA; (v) cohort, case-control, cross-sectional studies or any study evaluating health system performance; and iv) reported on one or more of the primary outcomes of interest:

1. Coverage of routine viral load monitoring for the eligible population at recommended time points.
2. Proportion of individuals from routine VL monitoring identified as failing treatment who (a) received EAC (b) were subsequently switched to second-line ART (c) achieved viral suppression.

Any case reports, reviews and mathematical models were excluded. After the title screen, the abstracts of articles possibly eligible for inclusion were screened based on the aforementioned criteria. The full texts of the remaining articles were obtained, with each being independently screened by AK for eligibility using the same criteria, and data was extracted using a standardized data extraction table. Screening and eligibility were independently verified by CI. Extracted information on the final selection of articles included: authors, study setting, population and year, VL monitoring coverage and cascade outcomes reported. Not all studies reported on each step of the cascade.

### Quality assessment

The Critical Appraisal Skills Programme quality assessment tool was used to assess the quality and risk of bias of the studies included for review [18]. The criteria assess the clarity of the study’s focus, whether participants were recruited in an acceptable way, if the primary outcome was accurately measured to minimise bias, and the implications of the study for local practice. The responses of ‘yes’, ‘can’t tell’, or ‘no’ to these questions, categorise the study’s risk of bias (Table S2).

### Data synthesis and statistical analysis

A narrative synthesis of selected studies was conducted. A meta-analysis of data was not suitable for this review, due to heterogeneity in the included studies arising from differences in monitoring timeframes, definition of VL suppression and study specific participant selection criteria.

## Results

**Fig. 2:**
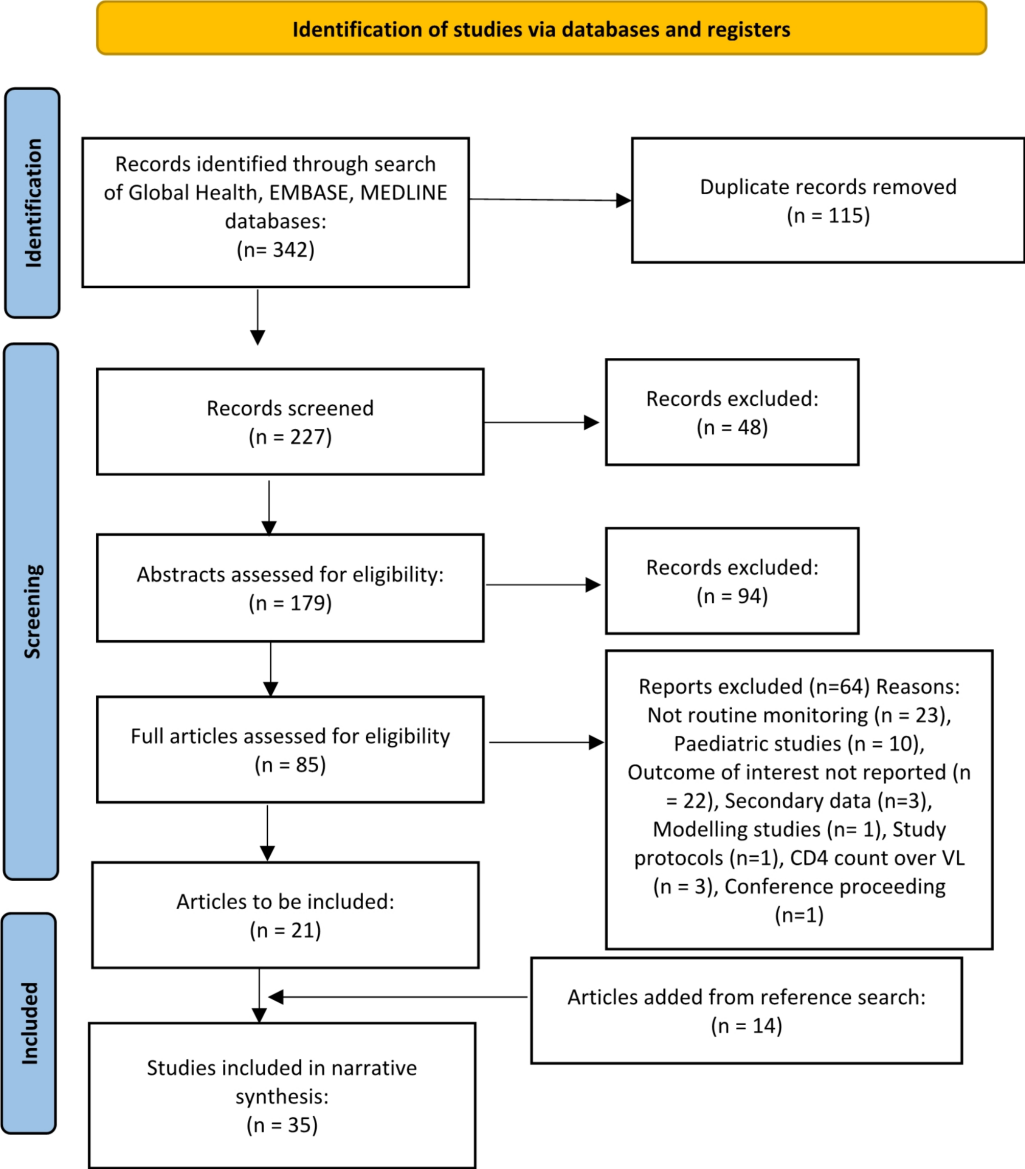
PRISMA flow chart of the study section process for articles published from 1st January 2004 to 9th August 2024 [17]

The initial search identified 342 papers; once duplicates were removed, 227 papers remained. The title screening process removed a further 48 papers unrelated to the outcome of interest. Screening of the abstracts left 85 articles for a full-text review, and of those, 21 met all inclusion criteria. A further 14 papers were included from a search of bibliographies, leaving 35 studies contributing to this systematic review (Fig. 2).

The 35 studies, involving a total of 874,927 participants, with 66.85% being female (excluding three studies [19–21], were carried out in 14 SSA countries: twelve in South Africa [19, 22–32], four in Rwanda [33–36], four in Uganda [37–40], three in Nigeria [41–43], three in Zimbabwe [21, 44, 45] two in Lesotho [11, 46], two from Zambia [20, 29] one from each of the following countries: Malawi [47], Cameroon [48], Mozambique [49], Tanzania [50], Swaziland (from here to be referred to as Eswatini^1^) [51], Kenya [44] and the Democratic Republic of Congo (DRC) [52]. For all of the studies, participants were PLHIV, seven studies assessed PLHIV initiating ART [23, 25, 29, 33, 36, 42, 43], six studies required participants to have been on ART for at least six months [34, 38, 39, 46, 49, 50], one study required being on ART for three months [47], two studies required being on ART for 12 months [31, 44], and one required being on ART for 18 months [48]. Two studies looked at participants on ART with a VL result of > 1000 copies/mL [24, 51], one study looked solely at participants on second-line ART [41], and the remaining studies did not have specific ART criteria [11, 19–22, 26–28, 30, 32, 35, 38, 40, 45, 52]. Twelve studies reported data on the VL monitoring and treatment outcomes at each step of the VL cascade of care apart from EAC [11, 20, 23, 25, 27, 34, 36, 44, 47–50]; only three papers studied had data on EAC in the cascade [21, 46, 51]. The remaining studies either focused on VL monitoring uptake [19, 26, 28–30, 32, 33, 38–40, 52] or other aspects of the cascade and 90-90-90 targets [22, 24, 31, 35, 37, 41–43, 45].

Out of 27 studies that reported on uptake of routine VL monitoring, a wide range of results were observed (Table 1). Rates of VL testing reported at facilities ranged from 24.3 − 99.7%, with certain programmes in Rwanda [33, 36], Malawi [34], Tanzania [50], Nigeria [43] and South Africa [26] achieving their 90-90-90 targets which were the relevant targets at the time the studies were conducted. Annual uptake of VL monitoring varied across countries, from 25.3% in Zimbabwe [44] to 94% in South Africa [26] (mean: 56%). Similarly, VL monitoring rates varied within countries, for example, in South Africa, VL uptake ranged from 34% in the sub-district of Hlabisa [25] and 94% in eThekwini metropolitan municipality [26], both in KwaZulu-Natal province. Four of the five programmes attaining testing rates of over 90% had received additional support, funding or evaluated interventions to improve the quality of ART and VL monitoring services [26, 33, 34, 43]. One study in the DRC demonstrated this difference by comparing testing rates in Médecins Sans Frontières (MSF) assisted hospitals with decentralised ART distribution clinics, and the rates of routine VL testing were 89% and 38.5% respectively [52]. However, MSF support does not always imply favourable results: a study from MSF-supported health centres in Mozambique only obtained VL testing rates of 40% [49], another in Zimbabwe achieving rates of 63% [21].

**Table 1:** Studies showing rates of VL testing and suppression

	Author, year	Study Setting (Sampling Year)	Study Population/Size	Age of Participants	Study Design	Primary Outcomes	Results of VL monitoring and cascade outcomes	VL monitoring in national guidance	Risk of bias
1	Opito et al., 2020	Eastern Uganda (2017–2018)	580 participants, 540 adults, newly diagnosed with HIV and initiating ART at TASO Tororo clinic (56.5% female)	Mean: 38.0 ± 13.1 years	Retrospective cohort	Uptake of ART under test and treat, 12-month retention, viral load monitoring rates and viral suppression	- 199/392 (50.8%) active in care monitored for VL - 184/199 (94.5%) virally suppressed (VL < 1000 copies/ml)	2015–2020: Yes [53]	Low risk
2	Ross et al., 2020	Rwanda (2018)	12,238 participants enrolled in HIV care at 10 health centres affiliated with CA-IeDEA (58% female)	15–24 years: 817 participants (8%) > 25 years: 9,393 participants (92%)	Cross-sectional	Being on ART, retained on ART (for > 2 post-ART clinic visits), available VL tests and viral suppression	- 10,200/12,238 (91%) had available VL results - 9331/12,238 (91%) virally suppressed (VL < 200 copies/ml)	2018: Yes [54]	Low risk
3	Nakalega et al., 2020	Uganda (2017)	414 participants receiving ART for at least 6 months, at 8 of the ART clinics in the Gomba district (60% female)	Median: 40 years (IQR 31–48)	Cross-sectional	To investigate factors for non-uptake of VL testing	- 276/414 (66%) had VL test	2015–2020: Yes [53]	At risk
4	Musengimana et al., 2022	Rwanda (2016–2018)	957 participants enrolled in HIV care in Rwinkwavu, Kirehe and Butaro district hospitals (and 43 affiliated health centres) (62.25% female)	15–24 years: 200 participants (20.9%) > 25 years: 757 participants (79.1%)	Retrospective cohort	Comparing clinical outcomes of patients with and without advanced HIV	- 342/957 (36%) received VL test within 9 months - 144/957 (15%) had virological failure at 12 months (VL > 200 copies/ml)	2009–2012: Yes [55]	Low risk
5	Chang et al., 2022	Nigeria (2018–2019)	541 participants initiating ART at Jos University Teaching Hospital and Comprehensive Health Centre Zamko (65.7% female)	Median: 36 years (IQR 30–43)	RCT	Investigating rates of viral suppression and retention in HIV services	- 302/541 (55.8%) had documented viral suppression at 12 months (VL < 1000 copies/ml) - 349/541 (64.5%) attended clinic visit at month 12 - Point-of-care arm had more documented VL results than standard-of-care arm	2017–2021: Yes [56]	At risk
6	Euvrad et al., 2019	South Africa (2015–2016)	22,991 participants receiving ART at healthcare facilities in Khayelitsha (72% female)	No data	Retrospective cohort	Looking at rates of viral suppression, completion of VL testing, and collection of VL data	- 18,450/22,991 (84%) had a timely VL test (within patient-specific window period around expected VL due date) - 16,420/18,450 (89%) had viral suppression (< 1000 copies/ml)	2007–2011: Yes [57]	At risk
7	Sunpath et al., 2018	South Africa (2016–2017)	864 participants receiving ART at 3 clinics in eThekwini, Kwa-Zulu Natal (no data on sex of participants)	No data	Cross-sectional	Assessing the implementation of the VL champion to upscale VL monitoring	- 547/864 (63%) average VL 6 months testing completion pre-implementation - 4,889/5,196 (94%) average 6 months VL testing completion post-implementation	2007–2011: Yes [57]	Low risk
8	Apollo et al., 2021	Zimbabwe (2004–2017)	392,832 participants receiving ART for 12 months, from 529 health facilities (63.8% female)	20–29 years: 41,168 participants (11.4%) > 30 years: 318,431 participants 318,431 (88.6%)	Retrospective cohort	Determining the number of patients receiving VL testing, with virological suppression, virological failure, VL outcomes after EAC and patients switched to 2nd line ART	- 90,898/359,609 (25.3%) had an annual VL test - 14,275/90,898 (15.7%) had an unsuppressed VL result (VL > 1000 copies/ml) - Other results include participants < 15 years of age	2003–2007: No [58] 2013–2017: Yes[59]	Low risk
9	Moudachirou et al., 2020	Democratic Republic of Congo (2015–2017)	337 participants receiving ART transferred from the hospital to ART distribution community centres (PODIs) in Kinshasa (72% female)	Median: 47 years (IQR 41–54)	Retrospective cohort	Assessing sustained viral suppression and retention in HIV care after patients transferred to community HIV and ART services	- 118/306 (38.5%) had a routine VL test at 12 months - 110/118 (93%) were virally suppressed (VL > 1000 copies/ml)	2015: Yes[60]	At risk
10	Le Roux et al., 2019	South Africa (2013–2014)	579 participants receiving ART at Zithulele Hospital or its 11 associated clinics (67.7% female)	Mean: 32 ± 13.4 years	Retrospective cohort	Assessing the treatment and monitoring rates between urban and rural areas of South Africa	- 480/579 (82.9%) had a timely VL test (between 60 and 210 days after ART initiation) - 536/579 (92.6) had a VL test within 12 months of ART initiation - 547/579 (94.5%) were virally suppressed (< 1000 copies/ml)	2007–2011: Yes [57]	Low risk
11	Tsondai et al., 2017	South Africa (2011–2014)	3216 participants across 100 adherence clubs within Cape Town (70% female)	Median: 36.4 years (IQR 32.5–41.6)	Observational cohort	Assessing the scale-up of adherence clubs across the Cape Town health district	- 2782/3216 (86.5%), 1563/1846 (84.7%), 490/615 (79.7%) had VL tests at 4 months, 16 months and 28 months respectively - 2697/2782 (96.9%), 1496/1563 (95.7%) and 461/490 (94.1%) were virally suppressed (< 400 copies/ml) at 4 months, 16 months and 28 months respectively	2007–2011: Yes [57]	At risk
12	Mshweshwe et al., 2020	South Africa (2017)	826 participants from 10 public sector health facilities in the Ekurhuleni District (63.9% female)	Median: 32 years (IQR27–39)	Retrospective cohort	Assessing same day initiation of ART during routine care delivery and retention in care	- 455/826 (64.1%) had VL tests at 6 months - 359/455 (79%) were virally suppressed (< 400 copies/ml)	2017–2022: Yes [61]	Low risk
13	Ruel et al., 2023	Kenya and Uganda (2019)	1549 participants from 28 clinics in rural western Kenya and southwestern Uganda (80.6% female)	Median: 21 years (IQR 19–23)	RCT	To evaluate the effect of a multilevel health system intervention in helping adolescents and young adults reach viral suppression	- 1425/1549 (92%) of participants had a viral load at 2 years follow up - 1196/1425 (83.9%) were virally suppressed at 2 years (< 400 copies/ml)	Kenya 2019: Yes [62] Uganda 2015–2020: Yes [53]	Low risk

**Table 2:** Studies investigating the failure cascade - repeat VL test, EAC and switch to second-line ART

	Author, year	Study Setting (Sampling Year)	Study Population/Size	Age of Participants	Study Design	Primary Outcomes	Results of VL monitoring and cascade outcomes	VL monitoring in national guidance	Risk of Bias
14	Glass et al., 2019	Lesotho (2015–2018)	24,948 participants receiving ART in the district of Butha-Buthe (1 district hospital and 11 rural clinics) (73% female)	Median: 41 years (IQR 33–52)	Prospective cohort	Describing the VL care cascade through follow-up VL testing and decision making based on VL result	- 9,949/24,948 had VL tests (39.9%) - 1028/9,949 (11%) had VL > 1000 copies/ml - 260/1028 (25%) were managed according to guidelines: 410/1028 (40%) had follow-up VL, 260/1028 (25%) resuppressed (VL < 1000 copies/ml) or switched to 2nd line ART	2014: Yes [63]	Low risk
15	Awungafac et al., 2018	Cameroon (2013–2015)	830 participants receiving ART for 18 months, at Limbe and Buea Regional Hospital treatment centres, Bamenda Regional Hospital and Bafoussam Regional Hospital (65% female)	Median: 40 years (IQR 34.5–47.5)	Retrospective cohort	Assessing uptake and utilization of VL tests for clinical decision making	- 201/830 (24.3%) had VL test - 0/201 (0%) had second VL in time on ART - 190/201 (94.5%) had viral suppression (VL < 1000 copies/ml) - 5/11 (45%) with VF were switched to 2nd line therapy	2015: Yes (but ART based on CD4) [64]	Low risk
16	Swannet et al., 2017	Mozambique (2014–2015)	43,579 participants receiving ART for 6 months, at 6 MSF supported health centres in Maputo city (68% female)	15–25 years: 2,220 participants (5.4%) > 25 years: 39,361 (94.6%)	Retrospective cohort	Assessing VL testing scale up through uptake of VL testing and follow-up	- 16,006/41,581 (38.5%) had VL test - 903/2,651 (34%) of those with an initial result > 3000 copies/ml had a follow-up VL test - 558/903 (61.8%) had VL > 3000 copies/ml	Data in Portuguese only	At risk
17	Onyedum et al., 2013	Nigeria (2012)	4229 participants on 1st line ART and 186 participants switched to 2nd line ART, at the HIV care and treatment centre of the University of Nigeria Teaching Hospital (59.1% female)	Mean: 41.8 ± 9.6 years	Retrospective cohort	Investigating reasons behind ART switch and cascade outcomes for those on 2nd line ART	- 186/4,229 (4.4%) switched to 2nd line ART - 147/186 (79%) switched due to virological failure on VL test (VL > 1000 copies/ml) - 93/144 (81.6%) had viral suppression on VL test after switch to 2nd line ART	2010–2015: Yes [65]	Low risk
18	Kehoe et al., 2020	South Africa (2011–2016)	8058 participants on ART and enrolled in adherence clubs in Khayelitsha, Cape Town (74% female)	Median: 39 years (IQR 34–45)	Longitudinal	Assessing proportion of patients with elevated VL result and cascade of care after confirmed virologic failure	- 7,136/8,058 (89%) had VL tests - 6,621/7,136 (93%) had viral suppression (VL < 400 copies/ml) - 150/441 (34%) with virological failure (VL > 1000 copies/ml) had no repeat VL test - 120/291 (41%) with VF were successfully resuppressed with EAC or treatment switch	2007–2011: Yes [57]	At risk
19	Rutstein et al., 2015	Malawi (not stated)	1498 participants on 1st line ART for 6 months, from 5 ART clinics in central and southern Malawi (70.3% female)	Mean: 42.1 years	Prospective cohort	Determining feasibility and effectiveness of dried blood spot for VL monitoring	- 1,494/1,498 (99.7%) had VL test - 1406/1498 (93.9%) were virally suppressed (VL > 5000 copies/ml) - 54/88 (61.4%) had confirmed virological failure with VL test - 50/54 (93%) initiated 2nd line ART	2014: Yes [66]	At risk
20	Keiser et al., 2011	South Africa (not stated)	18,706 participants initiating ART, in 4 ART programmes in Khayelitsha, Gugulethu, Tygerberg and Themba Lethu (65.7% female)	Median: 34 years (IQR 30–41)	Prospective cohort	Comparing rates of treatment switch with and without VL monitoring	- 8,892/14,258 (62.4%) of eligible patients at month 6 had VL test - 1,833/18,706 (9.8%) of total patients had switched to 2nd line ART at year 3 - 243/18,706 (1.3%) were on failing 1st line ART at year 3 (VL > 10,000 copies/ml)	2007–2011: Yes [57]	At risk
21	Sunpath et al., 2022	South Africa (2017)	116 participants receiving 1st line ART with VL > 1000 copies/ml, in 3 clinics in Durban (51.5% female)	Median: Pre-implementation 36 years (IQR 23–41) Post-implementation 35 years (IQR 30–39)	Before-after	Reporting the impact of the VL champion on the virological failure cascade of care	- Pre-implementation: 37/60 (61.7%) had a 2nd VL test 3/37 (8.3%) managed according to guidelines 4/37 (10.1%) switched to second line ART - Post-implementation: 29/56 (51.7%) had 2nd VL test 3/29 (10.7%) managed according to guidelines 5/29 (16.1%) changed to 2nd line ART	2017–2022: Yes [61]	At risk
22	Mnzava et al., 2022	Tanzania (2017–2020)	4454 participants receiving ART for 6 months in the districts of Kilombero and Ulanga, Morogoro region (69% female)	Median: 42 years (IQR 35–51)	Prospective cohort	Assessing the VL monitoring cascade of care and comparing turnaround times	- 4,238/4454 (95%) had a VL test - 3,683/4,238 (88%) had viral suppression (VL < 1000 copies/ml) - 425/494 (86%) had a follow-up VL after initial failure (VL > 1000 copies/ml) - 32/55 (58%) of those not already on 2nd -line ART were switched to 2nd line ART	2017–2022: Yes [67]	At risk
23	Iwuji et al., 2020	South Africa (2010–2016)	29,384 participants initiating ART in the sub-district of Hlabisa (69.9% female)	Median: 31 years (IQR 25–39)	Retrospective cohort	Determining if guidelines on management of virological failure are being implemented	- 9,861/24,199 (40.7%) 7,765/22,807 (34%) 4,334/16,965 (25.5%) had VL tests at 6, 12 and 24 months respectively - 2,135/19,582 (10%) had a VL > 1000 copies/ml - 658/2,135(30.8%) had a repeat VL test to confirm virological failure - 250/658 (38%) were resuppressed (VL < 1000 copies/ml) and 141/391 (36%) switched to 2nd line ART	2007–2011: Yes [57]	Low risk
24	Nicholas et al., 2019	Malawi (2013–2017)	21,400 participants receiving ART for 3 months in decentralised clinics and the district hospital of Chiradzulu (65% female)	Median: 38 years (IQR 31–46)	Retrospective cohort	Investigating outcomes from the first 4 years of routine VL monitoring using point-of-care testing	- 18,182/21,400 (85%) had a VL test - 16,150/18,182 (89%) had viral suppression after routine VL test - 1,281/1544 (83%) with virological failure (VL > 1000 copies/ml) had a follow-up VL test - 434/540 (80%) with confirmed VL failure on a 3rd VL test were switched to 2nd line ART - 275/347 (79%) were re-suppressed (VL < 1000 copies/ml) on repeat VL test after switch	2014: Yes [66]	At risk
25	Warrier et al., 2019	Zambia (2016–2018)	118,266 participants enrolled in HIV care with routine VL test result in 74 facilities across 3 Zambian provinces (no data on sex of participants)	No data	Retrospective cohort	Assessing each step of the failure cascade in order to help achieve the third “90” in the 90-90-90 goals	- 14,291/118,266 (12.1%) were virally unsuppressed after first VL test - 4,978/14,291 (9.2%) had a follow up VL within 90 days - 2,459/4,978 (49.4%) had virological failure (VL > 1000 copies/ml) - 720/2,459 (29.3%) were switched to 2nd line ART	2014–2016: Yes [68]	At risk
26	Labhardt et al., 2017	Lesotho (2014–2015)	138 participants receiving 1st line ART at 10 rural facilities in Lesotho for 6 months, with a VL > 80 copies/ml (65.9% female)	Median: 41.1 years (IQR 32.4–49.9)	Prospective cohort	Describing the outcomes of the failure cascade for patients with an unsuppressed VL	- 124/138 (90%) received EAC - 116/138 (84%) had a repeat VL test - 36/116 (31%) were virally re-suppressed (VL < 80 copies/ml) - 58/80 (73%) were switched to 2nd line ART	2014: Yes [63]	At risk
27	Etoori et al., 2022	Swaziland (2013–2015)	828 participants receiving ART with a VL > 1000 copies/ml, at 23 primary care clinics in the Shiselweni region, Swaziland (65.5% female)	Median: 35 years (IQR 29–44)	Retrospective cohort	Understanding where the gaps from along the VL cascade of care	- 288/828 (34.8%) received 3 sessions of EAC - 696/828 (84.1%) had a follow up VL test - 410/696 (58.9%) had virological failure (VL > 1000 copies/ml) - 120/278 (43.2%) were switched to 2nd line ART	2009–2014: No [69]	At risk
28	Hermans et al., 2020	South Africa (2007–2008)	104,719 participants receiving ART at 52 urban and rural facilities across 4 provinces of South Africa (67.6% female)	Median: 35.7 years (IQR 29.9–43.0)	Retrospective cohort	Assessing rates of virological suppression and the clinical management of viraemia	- 93,200/104,719 (89%) were virally suppressed at 12 months (VL < 1000 copies/ml) - 20,766/104,719 (19.8%) had virological failure at follow-up - 13,210/20,766 (63.6%) had a follow up VL test - 7,180/13,210 (54.4%) had virological failure (VL > 1000 copies/ml) - 1,872/4,510 (41.5%) were switched to 2nd line ART	2007–2011: Yes [57]	Low risk
29	Ntwali et al., 2019	Rwanda (2012–2016)	775 participants initiating ART from 1 public hospital and 1 health centre in the Northern Province of Rwanda (67.0% female)	Median: 34 years (IQR 27–41)	Retrospective cohort	Looking at the use of routine VL testing after installing a VL testing platform in the study district	- 510/547 (93.2%) participants had annual VL test - 451/510 (88.5%) had viral suppression (VL < 1000 copies/ml) - 103/117 (88%) had a follow up VL after initial elevated VL test - 26/41 (63.4%) switched to 2nd line ART	2009–2012: Yes [55]	Low risk
30	Nyagadza et al., 2019	Zimbabwe (2016–2017)	9456 participants from 10 sites in Manicaland Province (no data on sex of participants)	No data on total participants Median: 32 years (IQR 15–43) for those with a high VL result	Retrospective cohort	To disseminate lessons learned from Médecins Sans Frontier’s involvement with HIV VL testing scale-up in collaboration with the ministry of health	- 5966/9456 (63%) had a VL test after 6 months on ART - 5033/5966 (84%) were virally suppressed (< 1000 copies/ml) - 205/662 (31%) of virally unsuppressed patients with documentation had 1 documented EAC session - 96/201 (47.8%) had a second VL test - 69/96 (72%) were virally unsuppressed after the repeat VL test (> 1000 copies/ml) - 32/69 (46.4%) were switched to 2nd line ART	2013–2017: Yes [59]	At Risk

**Table 3:** Special category studies

	Author, year	Study Setting (Sampling Year)	Study Population/Size	Age of Participants	Study Design	Primary Outcomes	Results of VL monitoring and cascade outcomes	VL monitoring in national guidance	Risk of Bias
31	Woldesenbet et al., 2021	South Africa (2019)	8112 pregnant participants receiving ART for at least 3 months during pregnancy, or had initiated ART before pregnancy from 1589 public health facilities across South Africa (100% female)	Median: 26 years (IQR 22–31)	Cross-sectional	Determining the coverage of maternal viral load monitoring nationally, focussing on testing, documentation and suppression	- 6542/8112 (81.7%) received viral load testing at entry to antenatal care or at 3 months after ART initiation - 4277/8112 (74.1%) were virally suppressed (VL < 50 copies/ml) - 944/8112 (16.4%) had a VL between 50-1000 copies/ml)	2017–2022: Yes [61]	At Risk
32	Herce et al., 2020	South Africa and Zambia (2016–2017)	835 participants initiated ART at 10 correctional units in Johannesburg, Breede River and Lusaka (16% female)	Median: 32 years (IQR 28–28)	Prospective cohort	Assessment of feasibility of the universal test and treat intervention in correctional facilities and clinical outcomes for incarcerated people	- 269/346 (78%) had a VL test at 6 months - 262/269 (97%) were virally suppressed (< 1000 copies/ml) - (289 not then eligible for 6-month follow-up)	SA 2007–2011: Yes [57] Zambia 2014–2016: Yes [68]	At Risk
33	Nyakura et al., 2019)	Zimbabwe (2018–2019)	1112 pregnant participants receiving ART prior to pregnancy, or on ART for 3 months during pregnancy in public health facilities (3 hospitals and 25 rural health clinics) in the Mazowe district (100% female)	Mean: 30.3 years	Retrospective cohort	To determine the proportion undergoing VL testing and VL suppression up to 6 months after birth	- 354/1112 (31.8%) had a viral load test at first antenatal visit, or 3 months after ART initiation - 334/354 (94.4%) were virally suppressed (< 1000 copies/ml) - 13/20 (65%) had a repeat VL test	2013–2017: Yes [59]	At Risk
34	Namale et al., 2019	Uganda (2015–2016)	584 female sex worker participants receiving ART for at least 6 months at the Good Health for Women Project clinic south of Kampala (100% female)	Mean: 32.5 ± 6.5 years	Cross-sectional	Assessing prevalence of virological failure among female sex workers	- 432/584 (74.0%) had a VL test at 6 months - 394/432(91%) were virally suppressed (< 1000 copies/ml)	2015–2020: Yes [53]	At Risk
35	Nwosu et al., 2024	Nigeria (2016–2022)	34,976 female sex worker participants from the National HIV Key Populations program (100% female)	20–39 years: 26,599 (76%) participants	Cross-sectional	To examine factors associated with VL testing and suppression among FSWs in Nigeria	- 33,945/ (97.1%) had an initial VL test - 22,321/ (63.8%) had a repeat VL test at 90 days - 32,092/33,092 (94.5%) were virally suppressed (< 1000 copies/ml)	2017–2021: Yes [56]	At Risk

After an initially elevated VL test result, 17 studies reported on rates of follow-up VL testing to confirm virologic failure (Table 2). Follow-up VL monitoring results varied again across countries and study settings; however, in all settings, rates of follow-up VL testing were lower than for routine VL testing. Cameroon reported rates of follow-up VL to be 0% [48], the lowest of all countries included, and Rwanda performed the best, with rates of follow-up VL testing of 88%) [36]. Only three studies specifically investigated the uptake of EAC, the second step of the failure cascade. A study in Lesotho [46] demonstrated that 90% of patients with an initial elevated VL test result received EAC, 31% of whom were then virally re-suppressed; whereas in Eswatini [51] rates of EAC were lower at only 34.8%, of whom 41.1% were then re-suppressed on testing. In an MSF supported clinic in Zimbabwe [21], 31% of participants received 1 session of EAC, of which 47.3% had a repeat viral load, and 28% were virally supressed, suggesting retention in care to be a key barrier to be addressed. Other studies show rates of re-suppression, but do not show if this was following EAC or a switch to second-line ART.

The final step of the failure cascade, switching to second-line ART after confirmed virologic failure, was reported in 17 studies (Table 2). Rates of ART switch to second-line treatments are markedly lower across all countries and study settings. Rates ranged from 4.4% in Nigeria [41] to 93% in Malawi [34] (mean: 42% across all studies). Results of patients switched to second-line treatment varied within countries, for example in South Africa, treatment switch ranged from 9.8% [23] to 41.5% [27] (mean: 28.9%). Overall, studies with additional interventions targeted at improving ART services did not have better outcomes with regards to ART switches. For example, one study conducted by Sunpath et al. (2022) assessed the impact of the VL champion (a health-system strengthening role to improve care of patients with a raised VL result), and rate of ART switch was 16.1%, which is 25.4% lower than results from a study that collected data across four South African provinces, with no additional interventions or funding.

The final step of the VL monitoring cascade, and the aim of assessing ART and HIV services, is viral suppression. Viral suppression was reported after routine VL testing and also after switching to second-line ART or EAC. Studies that commented on rates of viral suppression during routine VL monitoring had a range of 55.8% [42]-97% (mean: 85.4% across studies). 13 studies from Uganda [37, 39], Rwanda [33], Cameroon [48], South Africa [22, 25, 29–31], Zambia [29], Malawi [34, 43] Nigeria [43], Zimbabwe [45] and DRC [52] had rates of viral suppression over 90% and, therefore, met the 90-90-90 targets. However, only the studies from Rwanda [33], Malawi [34], and Nigeria [43] also had monitoring rates over 90%, indicating that for the other studies, this level of viral suppression is not representative of the population being assessed. Programmatic data from Rwanda, Malawi and Nigeria indicate that these results are somewhat generalizable, with rates of viral suppression at 94% [70], 87% [71] and 82% [72] respectively. The same cannot be said for Uganda, South Africa and DRC, where programmatic data demonstrates rates of VL suppression are less favourable than the study results, with rates of 79% [73], 71% [74] and 77% [75] respectively. The remaining studies’ results are susceptible to survival and follow-up bias, as levels of repeat VL testing and retention in care are sub-optimal, and levels of viral suppression are therefore only attributable to those who are engaged in care services, with VL being observed and suppressed at earlier time points. For example, in Cameroon [48], from 24.3% having an initial VL test, there was 0% uptake in follow-up testing, however viral suppression was noted to be 94% at 12 months. This result does not account for those lost to follow-up, or those whose disease progressed to fatality and therefore may not be generalizable to the population. Only four papers assessed rates of VL monitoring and viral suppression after switch to second-line ART or EAC, and rates are lower across the studies, ranging from 25% [11] – 81.6% [41] (mean: 56.7%).

Three studies investigated VL monitoring amongst female sex workers [39, 43] and incarcerated individuals [29], some of the WHO’s defined key populations disproportionately affected by HIV [76], had a mean VL testing rate of 83% (Table 3). A study in Nigeria from the National HIV Key Populations Program [43] performed the best out of all the included studies with 97.1% having a VL test, and 94.5% achieving viral suppression. Both studies involving female sex workers involved programs aimed at increasing access to ART and providing comprehensive services to at risk groups. The study involving incarcerated individuals [29] achieved 97% viral suppression, however considering it had HIV testing and treatment services on site, only achieved 78% VL testing. Loss of follow-up due to participants changing or leaving correctional facilities was significant, and therefore results are not an accurate representation of the population. Two studies looked at pregnant women, both of which were not on track to meet UNAIDS goals.

### Quality assessment of included studies

The quality assessment demonstrated that all studies had clear primary outcomes and objectives; however, not all were clear in their recruitment of participants for the study, and validity of some study results may have been compromised in how the outcomes were measured. For example, some studies did not indicate when recruitment of participants and the conduct of the study itself took place [23, 34], making comparison of results difficult. For studies that used routine data but did not include loss to follow up, suboptimal quality of data collection and interpretation of final cohort results may have resulted in bias [23, 29, 51]. Overall, 14 studies out of 35 were assessed to be at low risk of bias (Table S2).

## Discussion

This systematic review synthesised viral load monitoring data for PLHIV from 14 countries in SSA. Just over half of these studies were published from 2020 onwards, coinciding with UNAIDS 90-90-90 targets aspirational deadline, indicating an increase in interest on research regarding the VL cascade and subsequent outcomes. Most studies were designed with the primary outcomes of investigating rates of VL monitoring and retention in care for PLHIV on ART, reporting on rates of viral suppression, and on outcomes from the cascade of care after confirmation of virological failure. Since the WHO recommended VL monitoring as the gold standard for monitoring disease progression and treatment efficacy [8], and the inception of “test and treat”, the demand for ART services and treatment monitoring will continue to increase. The need to audit and improve HIV service provision, as patients commence on life-long treatment is necessary [77]. Lack of government support and funding, inadequate infrastructure and access to resources, expense and lack of access to second-line and third-line ART, and clinicians’ failure to act on VL results, were common themes for suboptimal results, and consistent with findings from previous studies [5].

The results demonstrate significant gaps in the VL monitoring cascade both between countries and within countries, indicating the effect programmatic differences across regions can have on achieving national goals. One postulate for certain countries having significant differences in rates of VL monitoring is the urban-rural divide, where poor health outcomes in rural communities exacerbate poverty and inequality [78]. Rural areas are more likely to face inadequate access to resources, insufficient infrastructure, transportation difficulties and limited staffing [78, 79], alongside socio-economic constraints on patients that contribute to loss of follow-up and wasted appointments [80]. All are factors giving rise to suboptimal outcomes. Studies conducted in rural settings that had good rates of VL monitoring coverage, had decentralised care, and differentiated care within that. Examples include community-based models through adherence clubs in South Africa [22, 31]; testing via dried blood spots (DBS) in Malawi [34], which were then transported to laboratories; and point-of-care (POC) testing, allowing on-site interpretation of results in Tanzania and correctional facilities in South Africa and Zambia [29, 50].

These methods of decentralising HIV care focussed on cost-benefit and feasibility of implementation and should result in programme sustainability in the long-term; however, they have drawbacks. DBS use eliminates the need for trained medical personnel, as well as cold-chain transportation systems [81]; however, this delays treatment initiation or switches, as results take weeks to come back. POC testing, has the advantage of reducing the demand on central laboratories, hence treatment can in principle be initiated sooner. However, not all patients wait for the two hours turnaround time for results due socio-economic constraints and other competing livelihood priorities [80]. Asking patients to attend multiple repeat visits for confirmation of virologic failure and commencement of EAC not only delays decision making [81], but also increases the burden of seeking care [50], both of which are barriers to patient retainment and viral suppression. This indicates that timeliness of clinical decision making will help to improve treatment outcomes, reduce rates of advanced disease and prevent the accumulation of drug-resistant HIV [81].

In addition to decentralisation of testing services, studies with optimal coverage of VL monitoring also had additional support from non-governmental organisations such as MSF [22, 47, 51, 52], and had free HIV services for all. The financial challenges associated with the provision of HIV care and routine VL monitoring services is substantial, considering the majority of SSA countries are low income. Across SSA, 20% of governments’ total spending on health is on HIV services, of which 85% is from international funding, given that most SSA countries are unable to generate sufficient domestic public resources to cover the cost [82]. International funding is critical for the implementation of HIV monitoring and provision of services; however, it has declined by 6% since 2010 [83], and will continue to decline as health targets have continuously not been met, meaning it would take many more years of continuous funding for HIV to be eliminated in this region. Funding of HIV programmes should be focused on efficiency and integration into local health systems, so programmes can be sustainable, and continue long after funding has ceased. Health system strengthening will be important, focusing not only on providing the infrastructure and the resources, but also on health education to empower patients to be responsible for their disease management [81].

This review highlights suboptimal follow-up VL monitoring after virologic failure and switch to second-line ART, with no follow up testing at all in Cameroon [48], and rates of treatment switch as low as 4.4% in Nigeria [41]. Limited ART switch was attributed to issues of supply and demand, with insufficient supply of second-line ART, due to the increased demand, as scale-up of testing services resulted in an increased number of patients requiring second-line ART [41]. In addition to this, multiple studies [11, 27, 46, 47, 51] described clinicians’ reluctance to switch patients to second-line ART if they appeared clinically well or suspected treatment non-adherence, as these drugs are limited and costly. Delayed switching to second-line ART, can contribute to accumulation of drug resistance mutations, the incidence of which is increasing rapidly in SSA. The WHO reported that up to 24% of people initiating or re-initiating ART harbour drug resistance to non-nucleoside reverse transcriptase inhibitor-based first-line ART, hence the recommendation to switch to dolutegravir-based ART [84]. The benefits of resistance testing alongside VL monitoring are data collection on its prevalence and spread and informing ART regimens in treatment programmes based on the public health approach [85]. This, however, requires additional expenditure on testing facilities, training of staff, and accessibility of second and third-line treatment options. Many countries in SSA, including the majority of countries included in this review, have adopted dolutegravir-based ART as standard of care in accordance with WHO guidelines [86], due to its high genetic barrier to resistance and tolerability [86]. However, this was only introduced in SSA from 2018 onwards, after the data collection period for most studies included in this review [28, 40, 45]. Reports on performance of ART programmes that have adopted this new standard of care are now starting to emerge.

VL monitoring rates for pregnant women were suboptimal in studies included in this review. Every additional week of ART during the antenatal period, reduces mother to child transmission by 20% [87], and given that in Zimbabwe [45] VL monitoring rates in mothers at the first antenatal visit was only 31.8%, there is a lot of work that needs to be done to support new mothers and implement treatment and monitoring early. Studies involving sex workers [39, 43] performed better with a study in Nigeria [43] performing the best, achieving rates of VL monitoring of 97.1%. The National HIV Key Populations programme in Nigeria uses a venue-based sampling method to reach female sex workers across hotspots, such as brothels, nightclubs and bars; and provides them with a comprehensive and free HIV prevention services. The targeted approach adopted by this programme is yielding optimal results, and their methods should be investigated for components that can be scaled up more widely.

The recent systematic review conducted by Pham et al. across lower- and middle-income countries, shared many similar conclusions [5]. Key themes for suboptimal VL monitoring and suppression were insufficient access to testing, suboptimal follow-up testing, and a lack of action on confirmed virological failure with EAC and switch to second and third-line ART; all of which are consistent with the findings explored in this systematic review. The importance of timely ART switch and how prolonged treatment failure can impact levels of drug-resistance mutations were also explored by Pham et al. [5], with similar concerns around how this will impact countries with limited access to drug resistance testing. This further backs up the need for increased support in the scale-up of VL testing across SSA, further research to address barriers to retention in care, and improved sustainability for the longevity of treatment and monitoring programmes.

A strength of this systematic review was that each step of the “failure cascade” was reported on beyond VL monitoring. It included many different studies using different methods of VL monitoring, both urban and rural, plasma and DBS, countries where “treat all” has been implemented since 2014 and those just undergoing scale-up. Whilst this prohibited meta-analysis, it allowed comparisons to be made, and hypotheses to be formed on the best method of scale-up and sustainability of HIV treatment programmes.

## Limitations

The main limitation of our approach is that only peer-reviewed published studies in English were included, and so some important studies from grey literature, or those not published in English may have been omitted. Some limitations arise from the evidence included in the review. Firstly, there were no data from 32 out of 46 SSA countries, and only one study from the West geographical region, limiting generalisability in this region. Future funding for studies should be prioritized in West African countries, for a more accurate assessment and wider coverage of viral load monitoring to highlight gaps and inform interventions. Secondly, few studies reported on the proportion of PLHIV completing each step of the cascade, the steps of EAC, repeat VL tests and switching to second-line ART. These are important, as investigation alongside general VL monitoring, allowing targeted interventions to be put in place to improve them, if rates are found to be low. Finally, most studies only included data from PLHIV that had received an initial VL monitoring result, meaning rates of viral suppression are unlikely to reflect that of the general population of PLHIV due to omission of those lost-to-follow-up or who failed to return for VL monitoring tests.

## Conclusion

VL monitoring and management of virological failure are suboptimal in many SSA countries due to individual and health system-related challenges. Future research should investigate different methods of scale-up, allowing countries to learn from one-another and work together to achieve the revised UNAIDS 95-95-95 targets by 2030. It is clear from cited literature that decentralisation of care improves viral load monitoring making it accessible for those in rural areas with limited infrastructure, resources, and financial capabilities. Community-based models of care were also shown to improve HIV treatment outcomes. To eliminate HIV as a public health threat by 2030, the global health community must continue to support the scale-up of HIV treatment programmes by funding targeted interventions that will contribute to efficient and effective decentralised care; general health system strengthening that will enable sustainability of future programmes; and population education to empower PLHIV to be responsible for their health.

## Electronic supplementary material

Below is the link to the electronic supplementary material.

Supplementary Material 1

## Data Availability

Data is provided within the manuscript.

## Footnotes

1 Swaziland changed its name to Eswatini in 2018.

## Abbreviations

AIDS: Acquired immunodeficiency syndrome
ART: Antiretroviral therapy
DBS: Dried blood spot
DRC: Democratic Republic of Congo
EAC: Enhanced adherence counselling
HIV: Human immunodeficiency virus
MSF: Médecins Sans Frontières
PLHIV: People living with HIV
POC: Point–of–care
PRISMA: Preferred Reporting Item for Systematic Reviews and Meta–Analyses
SSA: Sub–Saharan Africa
UNAIDS: Joint United Nations Programme on HIV and AIDS
VL: Viral Load
WHO: World Health Organisation
